# Emotional exhaustion and traumatic stress among healthcare workers during the COVID-19 pandemic: Longitudinal changes and protective factors

**DOI:** 10.1371/journal.pone.0291650

**Published:** 2023-12-15

**Authors:** András Spányik, Dávid Simon, Adrien Rigó, Mark D. Griffiths, Zsolt Demetrovics

**Affiliations:** 1 Doctoral School of Psychology, ELTE Eötvös Loránd University, Budapest, Hungary; 2 Institute of Psychology at ELTE Eötvös Loránd University, Budapest, Hungary; 3 Faculty of Social Science, ELTE Eötvös Loránd University, Budapest, Hungary; 4 International Gaming Research Unit, Psychology Department, Nottingham Trent University, Nottingham, United Kingdom; 5 Centre of Excellence in Responsible Gaming at the University of Gibraltar, Gibraltar, Gibraltar; University of Hafr Al-Batin, SAUDI ARABIA

## Abstract

**Background:**

Healthcare workers (HCWs) are at high risk of experiencing work-related stress, burnout syndrome, and depression, especially during infectious disease outbreaks like COVID-19. Contributing factors include increased workload, lack of personal protective equipment, and inadequate support from the healthcare administration. Longitudinal studies have shown that the mental health status of HCWs has deteriorated over time. Social support and compassion satisfaction (CS) are protective factors that can mitigate adverse mental health effects. The present longitudinal study examined the mental health status of HCWs during the COVID-19 outbreak and aimed to identify potential predictors and protective factors.

**Methods:**

The study comprised 386 healthcare workers in Hungary and was conducted in two waves (T1 and T2) from January 2021 to January 2022. Participants completed an online survey including the Professional Quality of Life Scale, Maslach Burnout Inventory, demographic and work-related background factors. Statistical analyses included descriptive statistics, and a cross-lagged panel model (CLPM).

**Results:**

Frontline HCWs had higher levels of secondary traumatic stress (STS) and emotional exhaustion (EE) than non-frontline healthcare workers. Both groups experienced significant increases in these measures between T1 and T2. The CLPM indicated that EE had a significant lagged effect on STS among frontline workers, while STS had a significant lagged effect on EE among non-frontline workers. CS had a significant protective effect on both STS and EE in both groups.

**Conclusions:**

The findings suggest that CS protects EE and STS, particularly among frontline HCWs. The study also showed that different causative relationships exist between these factors among frontline and non-frontline HCWs, which underlines the possible cyclical relationship between the two depending on the circumstances. The results provide insights into the protective role of positive work experiences and the importance of considering the needs of both frontline and non-frontline HCWs in preventive intervention programs.

## Introduction

Numerous studies have shown that healthcare workers (HCWs) working with infected patients are at particular risk due to increased work stress during health emergencies and epidemics [1]. A specific form of work stress is secondary traumatic stress (STS) that is a psychological response to professional contacts with traumatized individuals [2]. Secondary traumatic stress has been shown to be closely related to burnout, especially emotional exhaustion (EE) [3]. EE is one of the three main symptoms of job burnout, and is a response to a range of work-related stressors [4]. On the other hand there are several factors that has a protective role in connection with STS and EE such as compassion satisfaction [5–7]. Relatedly, the prevalence of depression and anxiety can also rise as a result of persistently increased stress at work among HCWs, as seen from studies performed after the SARS (severe acute respiratory syndrome; 2003) and MERS (Middle East respiratory syndrome; 2012) epidemics [8, 9].

Burnout is often an underemphasized and frequently unrecognized problem among healthcare workers (HCWs), and is a precursor to additional mental health issues [10]. Its significance was emphasized in a review which reported that up to 60% of HCWs may be affected by this problem [10]. Additionally, according to the Medscape National Physician Burnout Suicide Report, the burnout rate among physicians in 2020 was 43% [11]. In addition to burnout, a recent systematic review indicated that HCWs experience a high prevalence of other mental health problems [12]. For example, among HCWs, the prevalence rates were 24% for depression, 30% for anxiety symptoms and 13% for post-traumatic stress disorder (PTSD) [12].

Based on a review which focused on previous infectious disease outbreaks, it was reported that in most of the cases that frontline HCWs had poorer overall mental health compared to non-frontline HCWs during infectious outbreaks [13]. However, two studies in the same review have reported opposite findings, indicating that frontline HCWs find working with infected patients less mentally burdensome. These latter findings may be explained by the fact that non-frontline HCWs are less accustomed to critical patient care or the treatment of infectious patients, and that during a crisis, there needs to be more time for these HCWs to adapt to working on frontline medical activities [13].

Since the outbreak of the coronavirus disease-2019 (COVID-19) pandemic, several studies have addressed the mental health challenges of HCWs. The results of the studies showed that the mental health of HCWs deteriorated significantly during the pandemic [14, 15]. According to a systematic review performed in 2021, the most common mental health issues among HCWs during the COVID-19 pandemic were PTSD, anxiety, depression, and distress [16].

Previous research has identified several factors underlying the mental health problems associated with the COVID-19 pandemic, such as increased workload, lack of protective equipment, and lack of support from the healthcare administration [17, 18]. Mental health problems were significantly more common among HCWs working in the COVID-19 frontline compared to their colleagues in different areas [19, 20]. However, a 2021 study highlighted that non-frontline HCWs also experienced increased psychological distress during the pandemic [21].

While several cross-sectional studies have been performed on the aforementioned issues, only a few longitudinal studies have examined the changes in the mental health status of HCWs [15]. However, the importance of such studies is essential because (i) PTSD symptoms, depression, and burnout often occur later in crises, and (ii) longitudinal studies are able to detect the different psychological effects of different types of pandemic waves [8, 9, 22]. The few longitudinal studies available have shown that the mental health status of HCWs has gradually deteriorated as the pandemic progressed [22, 23].

Secondary traumatic stress (STS) and emotional exhaustion (EE) have also been the focus of researchers in association with the mental health challenges of HCWs dealing with critically ill patients [24, 25]. Regarding EE, previous research has assumed a wide range of prolonged workplace stressors such as monotonous work, excessive time pressure, and conflict of responsibilities [26]. In contrast, in the development of STS, exposure to patients who experienced traumatic events or witnessed severe trauma of patients plays a significant role [27].

A systematic review of the prevalence of burnout syndrome among HCWs during the COVID-19 pandemic reported that approximately 50% of frontline workers suffered from EE, and that non-frontline HCWs also experienced EE [25]. According to another systematic review published at the beginning of the pandemic, the prevalence of STS among HCWs varied widely from 7.4% to 35% [28]. The relationship between STS and EE has been widely studied among workers who are exposed to trauma [3]. Previously, several cross-sectional studies have reported the co-occurrence of STS and EE, but the potential bi-directional or unidirectional characteristics of the relationships have not been shown [29]. A previous longitudinal cross-lagged study reported the direction of the relationship, whereby a higher level of job burnout led to higher level of STS [30]. This phenomenon is supported by the conservation of resources (COR) model, which argues, that workers who are exposed to continuous stressors are more vulnerable to traumatic events, such as STS [31].

On the other hand, several factors, such as clear organizational communication and a personal sense of control, have been identified as protective factors for improved mental health status among HCWs exposed to increased stress during the COVID-19 outbreak [32]. According to Schug et al. [33], higher levels of social support and optimism also have a countervailing effect on depression and generalized anxiety among HCWs. Many studies have emphasized the protective role of compassion satisfaction (CS) in association with adverse mental health effects such as anxiety or burnout syndrome [5–7]. CS is a protective mental health factor because it refers to the positive feelings HCWs have regarding caregiving [34]. CS is facilitated by the social esteem surrounding work, the work’s social value, and a good relationship with colleagues [6]. CS has a countervailing effect for both EE and compassion fatigue (CF) is associated with self-confidence and effective self-protective mechanisms [35, 36]. A recent meta-analysis showed that specific interventions (e.g., meditation, group support sessions, or mobile applications created for mental health prevention) can facilitate CS among HCWs [37]. According to a literature review examining the mental health effects of COVID-19 pandemic on HCWs, only 5% of studies examined CS and reported medium to high levels of CS among HCWs [38].

### Aim of the present study

The purpose of the present (longitudinal) study was to examine the mental health impact of the COVID-19 pandemic on HCWs. The central aim was to examine the effects of burnout and secondary traumatization, as well as the potential protective effects of job satisfaction. More specifically, based on the aforementioned literature, the present study examined the longitudinal changes in various mental health indicators (e.g., emotional exhaustion, traumatic stress) during the COVID-19 pandemic in Hungary and analyzed the influencing factors underlying the different patterns of change in the mental health status of frontline and non-frontline workers. The present longitudinal study examined the extent to which emotional exhaustion and secondary traumatization among HCWs changed between the second and fourth waves of the COVID-19 pandemic in Hungary. Another essential aim of the research was to examine the effect of compassion satisfaction (a positive psychological factor) on changes in STS and EE.

**Fig 1:**
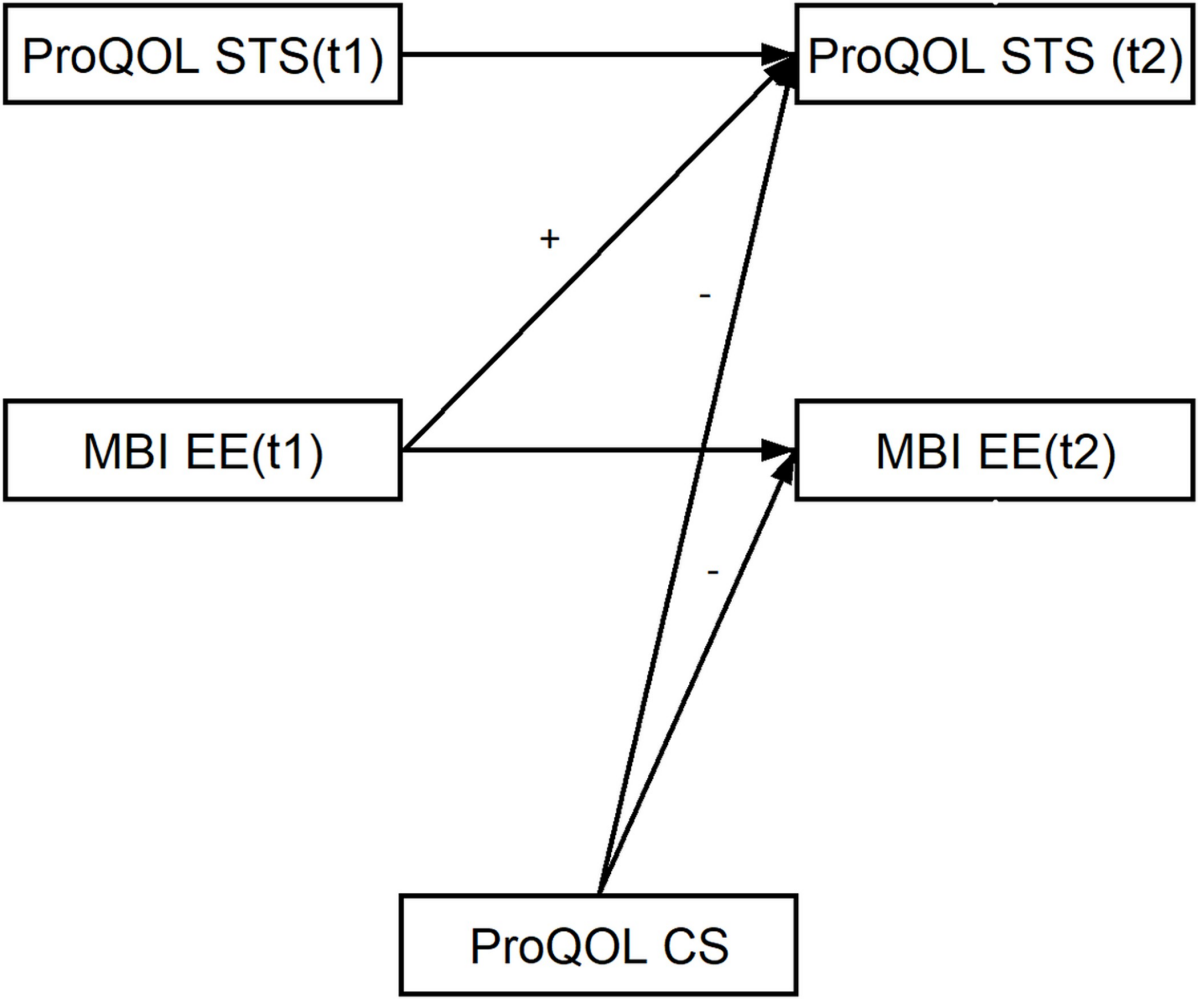
Hypothetical model of the relationships between emotional exhaustion, secondary traumatic stress and compassion satisfaction.

The hypotheses (H_s_) were that the: (i) level of emotional exhaustion and secondary traumatic stress would be higher among frontline HCWs compared to non-frontline HCWs (H_1_); (ii) emotional exhaustion and secondary traumatic stress would increase over time during the COVID-19 pandemic among healthcare workers (H_2_); (iii) compassion satisfaction would have a protective role in relation to emotional exhaustion and secondary traumatic stress (H_3_); and (iv) higher levels of emotional exhaustion would increase the level of secondary traumatic stress (according to the COR model) (H_4_) (Fig 1). In addition to the aforementioned hypotheses, the present study also investigated the disparities in temporal changes and causal relationships between emotional exhaustion and secondary traumatic stress among frontline and non-frontline HCWs. However, due to the absence of well-defined theoretical assumptions or prior research, these aspects were explored without specific hypotheses.

## Methods

The data collection took place between January 14, 2021 and January 19, 2022. During this period, a total of 1,022,338 people infected with COVID-19 were registered in Hungary. During the study period, the daily average number of individuals who were treated for COVID-19 in hospitals was 3,840 (SD = 3,564) (koronavirus.gov.hu). During the period of the present study, the number of hospitalized patients per million people was 395 (SD = 366), but the maximum number of hospitalized patients reached 1290 individuals per million. In this period, the number of hospital beds per one million people in Hungary was 4,271. Based on these figures, the extra workload of hospitals exceeded 30% during the most critical period [39].

### Procedure

The study was approved by the ELTE Eötvös Loránd University Research Ethics Committee. An online survey was developed using Qualtrics and a short description of the study was sent to members of the Hungarian Medical Chamber (HMC) and Hungarian Chamber of Health Professionals (HCHP) along with a link to the survey. Participants provided informed and voluntary written consent to participate in the study (through the survey system) and had the option to withdraw from the study at any time. The first data collection period of the study occurred between January 14 and March 6, 2021, during the second wave of the COVID-19 pandemic in Hungary (Time 1; T1). The second data collection period took place between November 26, 2021, and January 19, 2022, prior to the onset of the fifth wave of the COVID-19 pandemic in Hungary (Time 2; T2). Pseudonyms were used to match responses in the first and second waves. All participants were offered psychological aid, which was provided by a cooperating treatment service provider.

### Participants

The study comprised active healthcare workers from Hungary as participants. During the first wave of the research, 3321 surveys were started. To be eligible for inclusion, participants needed to complete at least 90% of the survey. A total of 2260 participants met the inclusion criteria in the first wave of the study. Of these, 340 agreed to participate in the second wave of the study. The sample comprised 79.9% females and 20.1% males, with ages ranging from 20 to 87 years (M = 45.91 years, SD = 13.48).

### Measures

#### Secondary traumatic stress

Work-related stress was assessed using the Secondary Traumatic Stress element of the Compassion Fatigue subscale of the Professional Quality of Life Scale (5^th^ version) (PQL STS), developed as part of a tool assessing the quality of life among caregiving professionals [34] (Hungarian version [40]). The ProQL STS comprises 10 Likert-type items. Each item (e.g., *“I find it difficult to separate my personal life from my life as a helper”*) is scored on a five-point scale from 1 (*never*) to 5 (*very often*) (scale range: 10–50; Cronbach alpha at T1 was .87, and for T2 was .85).

#### Compassion satisfaction

Work-related satisfaction was assessed in the second data collection period using the Compassion Satisfaction subscale of the Professional Quality of Life Scale (5^th^ version) (PQL CS) [34] (Hungarian version [40]). The subscale contains ten items related to the satisfaction dimension of a helping occupation. Each item (e.g., *“I get satisfaction from being able to help people”*) is scored on a five-point scale from 1 (*never*) to 5 (*very often*) (scale range: 10–50; Cronbach alpha at T2 was .92).

#### Emotional exhaustion

The emotional exhaustion factor of burnout was assessed by the Emotional Exhaustion subscale of the Maslach Burnout Inventory for Human Service Survey (MBI EE) [41] (Hungarian version [42]). The original version of the MBI EE subscale comprised nine items assessing the frequency of specific work-related feelings. Each item (e.g., *“Feel emotionally drained from work”*) is scored on a seven-point scale from 0 (*never*) to 6 (*every day*). During the evaluation of the Hungarian version, Item 14 did not fit in the MBI EE subscale, therefore it was omitted (scale range: 0–48; Cronbach alpha at T1 was .94, at T2 was .95).

### Statistical analysis

Descriptive statistics were calculated for all study variables (i.e., means, standard deviations, skewness, kurtosis). Paired sample student *t*-tests were used to examine the difference between study variables at T1 and T2. All variables were considered nearly normally distributed if skewness and kurtosis were in the range of +/-2 [43]. Skewness and kurtosis remained between -1.14 and +0.60 for all investigated variables in the present study. Cronbach’s α reliability estimation was conducted for psychometric scales. Correlation analysis was conducted by computing Pearson’s correlation coefficients with two-tailed significance tests. A *p* < .05 significance level was used for all statistical tests. SPSS v.23 was used for the descriptive statistics, reliability, and correlation analysis.

The research questions were investigated by cross-lagged panel model (CLPM) using structural equation modeling (SEM). The estimation method was selected according to the normality check of the aforementioned participating variables (maximum likelihood estimation with missing values). Stability and reciprocity, as well as ProQoL STS and MBI EE as antecedent models, were assessed using age as a control variable. The goodness of fit of the model was tested using likelihood ratio tests (model versus baseline, model versus saturated), root mean square error approximation (RMSEA), comparative fit index (CFI), Tucker-Lewis index (TLI), the goodness of fit index (GFI). A model is considered to fit well if RMSEA<0.06, TLI, CFI >0.95, and GFI>0.90 [44] and χ^2^/df under 2 (if chi-square test shows a significant difference from the saturated model) [45, 46]. The final model was selected by using a likelihood ratio test on embedded models. The final CLPM model was amended by ProQoL CS, and further multigroup analyses were conducted for frontline HCWs and non-frontline HCWs. Standardized coefficients and equation-level goodness of fit (R^2^) were also calculated in connection with the latter model. Stata 14 was used for all calculations for the SEM models.

## Results

### Descriptive statistics and preliminary analyses

In relation to ProQoL STS and EE, larger values were obtained from frontline HCWs compared to non-frontline HCWs (Table 1). This result supported H_1_ There was a significant increase between T1 and T2 among both groups with similar effect sizes (Cohen’s *d* 0.30 and 0.23, respectively). This result supported H_2_. Regarding MBI EE, similar differences and partially similar changes were found. Higher values were obtained among frontline HCWs compared to non-frontline HCWs.

**Table 1:** Comparative descriptive statistics of the study variables in the first and second waves of the COVID-19 pandemic among all respondents and subgroups of frontline healthcare workers and non-frontline healthcare workers.

		T1					T2					
Variables		N	M	SD	Skew.	Kurt.	n	M	SD	Skew.	Kurt.	Cohen-d
Secondary traumatic stress (ProQoL STS)	All	334	23.30_a_	7.28	.25	-.55	301	25.35_b_	7.52	0.33	-0.20	0.28
	Frontline	133	24.70_aa_	6.66	-.07	-.32	96	26.90_ab_	8.11	-0.09	0.05	0.30
	Non-frontline	184	22.70_ba_	7.50	.47	-.40	148	24.32_bb_	7.00	0.60	0.12	0.23
Emotional exhaustion (MBI EE)	All	336	24.61_a_	12.84	-.08	-.94	301	27.68_b_	13.84	-0.27	-1.12	0.23
	Frontline	134	28.13_aa_	11.45	-.27	-.76	96	29.54_aa_	13.11	-0.40	-0.90	0.11
	Non-frontline	185	22.82_ba_	13.07	.05	-.90	148	25.84_bb_	13.89	-0.15	-1.14	0.22
Compassion satisfaction (ProQoL CS)	All						300	35.40_b_	7.27	-0.38	-0.34	
	Frontline						96	34.68_a0b_	7.65	-0.44	-0.40	
	Non-frontline						148	36.29_a0b_	7.32	-0.44	-0.20	

Note. The sum of the number of frontline and non-frontline workers is not equal to the number of all participants due to missing information about working place. Means for frontline and non-frontline groups in the same column not sharing the same first subscript were significantly different tested by two-sided independent sample *t*-test, while means for first and second waves in the same row not sharing the same second subscript are significantly different tested by two-sided paired sample *t-*test (in both cases at *p* < .05). Changes over time were only assessed in where there were full sample means. ProQol STS: Professional Quality of Life Scale, Secondary traumatic stress component of Compassion Fatigue subscale; ProQol CS: Professional Quality of Life Scale, Compassion Satisfaction subscale; MBI EE: Maslach Burnout Inventory for Human Service Survey, Emotional Exhaustion subscale; Skew = Skewness; Kurt = Kurtosis

Table 2 provides Pearson correlations between all study variables. According to power analysis, a 0.17 correlation in the population can be detected with a type II error level of .10. Most correlations were found to be significant except for most correlations of occupation. All correlations between secondary traumatic stress, emotional exhaustion, and compassion satisfaction were medium to high and significant (between .33 and .64), except for the correlation between secondary traumatic stress at T1 and compassion satisfaction at T2. Correlations between secondary traumatic stress and emotional exhaustion were positive, while the correlations between compassion satisfaction and both secondary traumatic stress and emotional exhaustion were negative. Age was correlated with all psychometric scores significantly with moderate strength (between .20 and .42). Gender was weakly or not at all correlated with psychometric scales (between -.03 and .16). Significant positive weak correlations were found between frontline work and both secondary traumatic stress and emotional exhaustion, but it was not correlated with compassion satisfaction.

**Table 2:** Descriptive statistics and Pearson correlations for study variables.

Variables	n	M	SD	1	2	3	4	5	6	7	8	9	
1. Age	386	45.19	13.32	-									
2. Gender^a^	386	01.80	00.40	-.11^**^	-								
3. Occupation^b^	374	01.39	00.49	-.12^**^	.20^**^	-							
4. Frontline^c^	320	00.42	00.49	-.43^**^	-.08^**^	.01^**^	-						
5. Secondary traumatic stress, T1 (ProQoL STS)	334	23.30	07.28	-.21^**^	.14^**^	.04^**^	.14^**^	-					
6. Emotional exhaustion, T1 (MBI EE)	336	24.61	12.84	-.42^**^	.11^**^	.05^**^	.21^**^	.58^**^	-				
7. Secondary traumatic stress, T2 (ProQoL STS)	301	25.35	07.52	-.20^**^	.16^**^	.10^**^	.17^**^	.53^**^	.45^**^	-			
8. Emotional exhaustion, T2 (MBI EE)	301	27.68	13.84	-.41^**^	.15^**^	.04^**^	.13^**^	.39^**^	.64^**^	.56^**^	-		
9. Compassion satisfaction, T2 (ProQoL CS)	300	35.40	07.27	.29^**^	-.03^**^	.02^**^	-.11^**^	-.19^**^	-.44^**^	-.33^**^	-.57^**^	-	

Note.

^a^ 1 = male 2 = female

^b^ 1 = doctors 2 = other HCWs

^c^ 0 = not working in the frontline 1 = working in the frontline

^*^ *p* < .05^,^

^**^p < .01

ProQol STS: Professional Quality of Life Scale, Secondary traumatic stress component of Compassion Fatigue subscale; ProQol CS: Professional Quality of Life Scale, Compassion Satisfaction subscale; MBI EE: Maslach Burnout Inventory for Human Service Survey, Emotional Exhaustion subscale.

### Cross-lagged panel models

A maximum likelihood model with missing values was used for fitting the model because all criteria were met (Table 3). Neither the stability model (m1) nor secondary traumatic stress as an antecedent model (m2) fitted properly according to the chi-square test. The LR (likelihood ratio) test showed no significant increase in fit (*p* = .064). However, emotional exhaustion as an antecedent model (m3), showed a significant increase in fit compared to the stability model (*p* < .001) and yielded to perfect fit according to all fit statistics. As the reciprocity model (m4) did not show further significant improvement in fit according to the LR test (*p* = .389), further analysis was based on this model. These results partially supported H_4_. The inclusion of compassion satisfaction in the model (m5) resulted in a model that was fit according to all fit statistics.

**Table 3:** Fit statistics for the structural equation models.

Model (model number)	*n*	*χ* ^2^	*Df*	*p*	CFI	TLI	GFI	RMSEA	Model AIC	Model comparison	Δχ2(p)
Stability model (m1)	386	16.12	3	.001	.96	.90	.95	.106 (90% CI: .059, .160)	12074.2	-	-
Secondary traumatic stress as antecedent (m2)	386	12.69	2	.002	.97	.88	.96	.118 (90% CI: .062, .183)	12072.8	m1-m2	3.42 (.064)
Emotional exhaustion as antecedent (m3)	386	0.74	2	.689	1.00	1.00	.99	.000 (90% CI: .000, .075)	12060.8	m1-m3	15.37 (.000)
Reciprocity model (m4)	386	0.00	1	.965	1.00	1.00	.99	.000 (90% CI: .000, .000)	12062.1	m3-m4	0.74 (.389)
Emotional exhaustion as an antecedent with compassion satisfaction (m5)	386	2.42	2	.326	.99	.99	.99	.018 (90% CI: .000, .104)	13989.6	-	-
Emotional exhaustion as an antecedent with compassion satisfaction (frontline vs. non-frontline; m6)	320	5.15	4	.272	.99	.98	.98	.042 (90% CI: .000, .133)	11997.9	-	-
Reciprocity model with compassion satisfaction (m7)	320	0.137	1	.711	1.00	1.00	.99	.000 (90% CI: .000, .097)	13989.5	-	-
Reciprocity model with compassion satisfaction (frontline vs. non-frontline; m8)	320	0.273	2	.873	1.00	1.00	.99	.000 (90% CI: .000, .079)	11997.0	-	-

**Fig 2:**
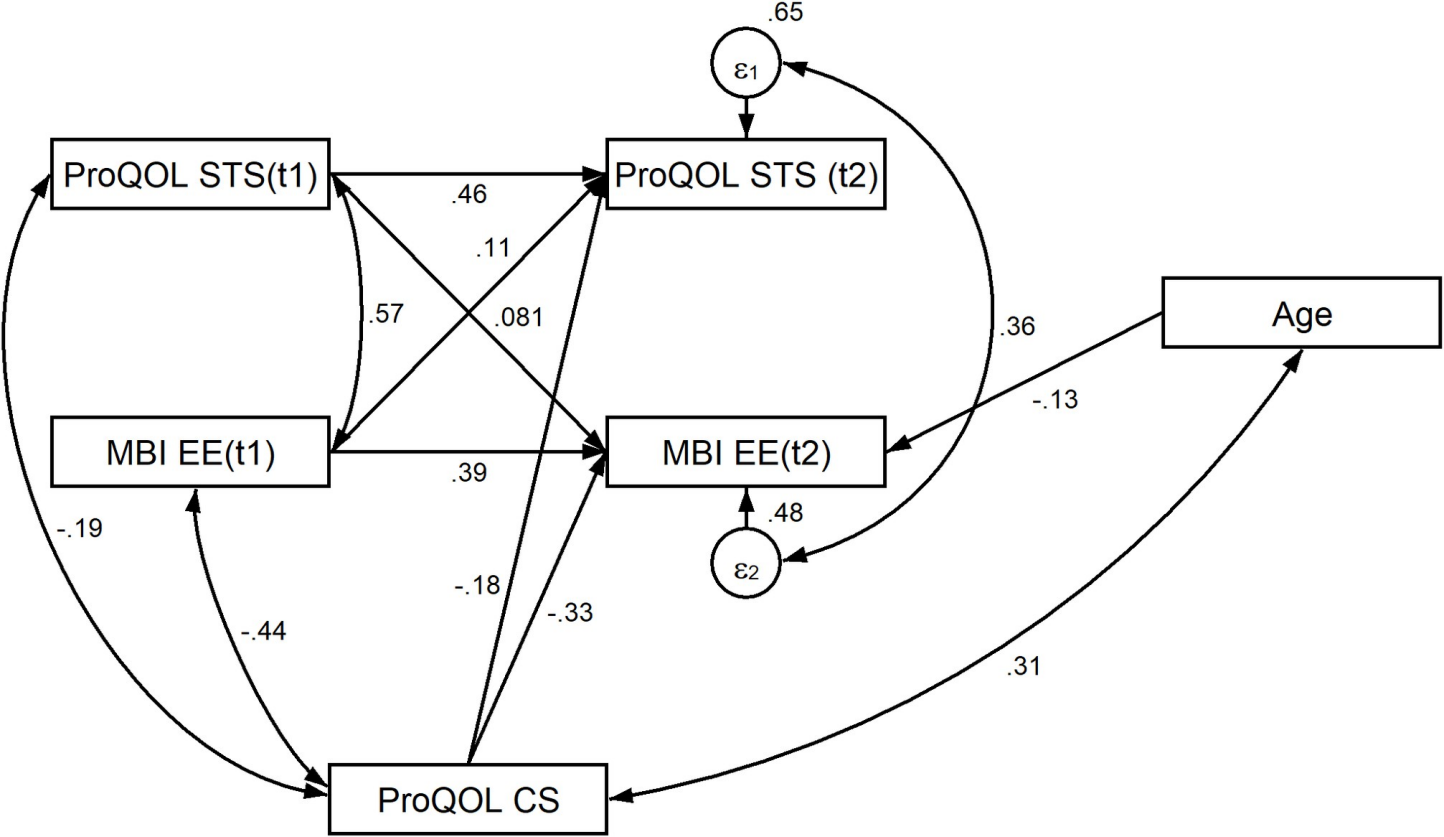
Reciprocity cross-lagged model (m7) between burnout and secondary traumatic stress with the effect of compassion satisfaction among healthcare workers. Note. ProQOL is Professional Quality of Life Scale, ProQOL STS is Secondary Traumatic Stress element of the Compassion Fatigue subscale of ProQOL, ProQOL CS is Compassion Satisfaction subscale of ProQOL, MBI EE is Emotional Exhaustion subscale of the Maslach Burnout Inventory for Human Service Survey.

The multigroup model for frontline and non-frontline groups (m6) also fitted well. The model for the frontline group (m6) was stable because there were no significant effects found between emotional exhaustion at T1 and secondary traumatic stress in T2 (*p* = .650) or between secondary traumatic stress at T1 and emotional exhaustion in T2 (*p* = .074). However, the lack of significance in the latter case could be due to a lower sample size. On the contrary, in the case of non-frontline HCWs, emotional exhaustion at T1 had a significant effect on secondary traumatic stress at T2 (*p* < .001). Consequently, further analysis of the reciprocity model with the inclusion of compassion satisfaction for the whole sample (m7) was conducted (Fig 2). This model, as well as the multigroup model for frontline and non-frontline HCW groups (m8) fitted well according to all fit statistics.

In the reciprocity model with compassion satisfaction for the whole sample, neither of the cross-lagged effects were significant (Table 4). However, compassion satisfaction had a significant negative effect on emotional exhaustion and secondary traumatic stress. Moreover, in the case of multigroup analysis, among non-frontline HCWs, secondary traumatic stress had a significant lagged effect on emotional exhaustion (.15; *p* = .025), but emotional exhaustion had no significant lagged effect on secondary traumatic stress (-.03; *p* = .710). On the contrary, among frontline HCWs, emotional exhaustion had a significant lagged effect on secondary traumatic stress (.29; *p* = .004), but secondary traumatic stress had no significant lagged effect on emotional exhaustion (-.01; *p* = .845). Compassion satisfaction had a significant negative effect in both groups on both emotional exhaustion and secondary traumatic stress, but the effect size was largest among frontline HCWs in the case of emotional exhaustion (-.41; *p* < .001) compared to non-frontline HCWs (-.29; *p* < .001) and on secondary traumatic stress among both frontline HCWs (-.21; *p* = .031) and among non-frontline HCWs (-.15; *p* = .045). These results supported H_3._

**Table 4:** Reciprocity cross-lagged model between burnout and secondary traumatic stress with the effect of compassion satisfaction among healthcare workers.

		All HCWs (m7)			Frontline (m8)			Non-frontline (m8)		
		Std. Coef.	z	*p*>|z|	Std. Coef.	Z	*p*>|z|	Std. Coef.	z	*p*>|z|
Emotional exhaustion (T2)	Emotional exhaustion (t1)	.38	6.62	.000	.37	4.08	.000	.36	4.74	.000
	Secondary traumatic stress (T1)	.08	1.46	.145	-.01	-0.20	.845	.15	2.24	.025
	Compassion satisfaction	-.35	-7.96	.000	-.41	-5.29	.000	-.29	-4.79	.000
	*Age*	-.11	-2.66	.008	-.11	-1.55	.121	-.19	-3.10	.002
Secondary traumatic stress (T2)	Secondary traumatic stress (T1)	.43	7.45	.000	.25	2.57	.010	.57	7.90	.000
	Emotional exhaustion (T1)	.12	1.95	.052	.29	2.90	.004	-.03	-0.37	.710
	Compassion satisfaction	-.19	-3.46	.001	-.21	-2.16	.031	-.15	-2.01	.045

## Discussion

The present study examined longitudinal changes in mental health indicators among healthcare workers (HCWs) during the COVID-19 pandemic in Hungary, analyzing factors influencing different pathways of mental health status by using cross-lagged panel models. Additionally, the study investigated the impact of a positive psychological factor, compassion satisfaction (CS), on changes in emotional exhaustion and secondary traumatization of HCWs during the second to fourth waves of the pandemic in Hungary.

The level of both STS and EE were found to be higher among frontline workers compared to non-frontline workers in both T1 and T2. This result supported H_1_ and concurs with previous studies highlighting the negative effect on mental health of HCWs working with infected individuals [1, 14, 15, 47].

The findings of the present study supported H_2_ by finding a significant increase in both secondary traumatic stress and emotional exhaustion among HCWs between T1 and T2, aligning with previous longitudinal studies indicating a gradual deterioration in the mental health status of HCWs during the pandemic [22, 23]. However, it is important to consider these findings in the context of a recent systematic review [47] which evaluated 45 studies. The review noted that although the prevalence of burnout among individuals in research studies decreased over time, it remained high among healthcare workers. Studies conducted during the early pandemic period reported a burnout prevalence rate of 60.7%, which decreased to 49.3% during the late pandemic period [47].

In the present study, when comparing the changes among frontline and non-frontline HCWs, there was a similar significant increase in secondary traumatic stress between T1 and T2 among both groups, whereas the change in the level of emotional exhaustion was only significant among non-frontline HCWs. This latter finding might be due to CS’s more substantive protecting effect on EE among frontline HCWs compared to non-frontline HCWs. The weak causative relationship between EE and STS disappeared with the inclusion of CS, which can be interpreted as further support for the protective role of CS suggested by many previous studies [5–7].

The effect of CS was found to be protective both in case of STS and EE among HCWs in general and in both subgroups which supports H_3_. The protective role of CS has been investigated in many other studies. Tremblay and Messervey [47] found a significant association between experiences in working with patients and the balance of compassion satisfaction and compassion fatigue reported by healthcare professionals. This indicated that those who reported more positive experiences with their patients tended to have a more favorable balance of compassion satisfaction and compassion fatigue (CF). Less research has addressed the role of CS as a protective factor against STS and CF. However, Cummings et al. [48] found evidence of a potential protective effect of CS on CF and STS. This highlights the importance of considering the positive dimensions of healthcare professionals’ work experiences in preventing and managing stress-related outcomes. Research also suggests that CS may have a buffering effect on the relationship between high job demands and job strain, potentially contributing to positive individual and organizational outcomes [49].

Comparing frontline and non-frontline HCWs, different causative relationships were found in the applied cross-lagged panel models. While among frontline HCWs, higher emotional exhaustion in T1 appeared to increase secondary traumatic stress in T2, among non-frontline HCWs, the causative relationship appeared to be the opposite. These results partially supported H_4_. The causative relationship among frontline HCWs concurs with the findings of two independent longitudinal studies by Shoji et al [30]. Together, the results underline the possible circular relationship between the two factors depending on the circumstances. According to Vagni et al. [50], an explanation of the differences could be that frontline HCWs during the COVID-19 pandemic performed their work in the environment that they were familiar with and under the usual conditions, while for the non-frontline HCWs, conditions were very different from the usual conditions at their workplace, and the frontline HCW’s work environment was inherently safer for both the caregivers and the patients. The nearly one-year stressful period for healthcare workers in 2021, during the highest peaks of the COVID-19 pandemic, resulted in different dynamics in secondary traumatic stress and emotional exhaustion and their relationship among frontline HCWs and non-frontline HCWs.

The study has some limitations that warrant consideration when interpreting the results. The sample size for the longitudinal analyses at T2 was relatively small, even though all members of the HMC and HCHP were given the opportunity to participate, therefore it was not possible to assess heterogeneity of HCWs in the model. Additionally, convenience sampling was used to recruit participants, which may limit the generalizability of the findings. The data collected were all self-report, which is known to be subject to various biases. The 10-month interval between the two waves of the research may not have been sufficient to capture changes in specific mental factors, although the findings were supported by previous studies. The survey was administered online, which constrained the length of the survey and prevented the assessment of other important factors, such as social support, an analysis of needs, and detailed questions regarding working conditions. Future research should address these gaps by conducting face-to-face studies with larger sample sizes and incorporating the aforementioned factors. Lastly, future research could benefit from an in-depth understanding of the psychological mechanisms underlying the findings, which could be achieved through further semi-structured interviews.

Considering the protective effect of CS on EE, preventive intervention programs for good mental health among HCWs should prioritize the enhancement of CS, as aforementioned. Various approaches, such as relaxation techniques, mindfulness practices, specific mobile applications, and group support sessions, have demonstrated a protective effect on adverse mental health outcomes by strengthening CS [31]. Based on the present study’s findings, decision-makers should also consider the increased vulnerability of non-frontline HCWs. To further support the importance of acquiring adequate coping strategies, a previous review emphasized the necessity of training workers to adopt appropriate coping styles as an essential component of prevention strategies to reduce the incidence of burnout [51].

The present study’s findings contribute to a more differentiated understanding of the different needs of HCWs working in distinct fields. Further studies are needed to gain a deeper understanding of the underlying factors and diverse support needs among the heterogeneous groups of HCWs observed in the present study.

The study also contributes to the theoretical understanding of the protective role of coping strategies and the dynamic relationship between emotional exhaustion and secondary traumatic stress, providing insights for future research and the development of effective interventions for healthcare professionals.

Additional research is needed to better understand the distinct experiences of burnout between frontline and non-frontline workers HCWs, and to explore the complex and dynamic relationship between emotional exhaustion and secondary traumatic stress among these two groups of HCWs. Such studies would contribute to advancing knowledge in the field and provide valuable insights for developing tailored interventions and support systems for healthcare professionals. As shown in the few previous studies [13], the greater resistance to emotional exhaustion observed in the case of frontline HCWs and the recognition of the significant protective effect of compassion satisfaction could contribute to creating more differentiated prevention programs.

## Conclusion

The present longitudinal study provides a comprehensive overview of the deteriorating mental health status among HCWs during the COVID-19 pandemic, and highlights the varying mental states among healthcare groups working in different areas. The other important aspect of the study is the examination of protective factors influencing the mental health status of HCWs.

Frontline HCWs attained higher scores in both STS and EE and both subgroups, and these scores increased over time. However, for EE, a significant increase was observed only among non-frontline HCWs. The possible explanation for the varying strength of the effect observed for EE might be due to the protective influence of CS among frontline workers. An additional explanation for the potential protective role of CS could be due to the attenuation of the weak causative effect between EE and CS. The distinct causative relationships found among frontline and non-frontline workers, as shown by the cross-lagged panel, also lend support to the protective role of CS.

The research findings will help contribute to designing targeted positive psychological healthcare interventions and draw attention to the poor mental state prevailing among HCWs, further exacerbated by the COVID-19 pandemic. Future research should further examine the experiences of burnout among frontline and non-frontline HCWs and explore the dynamic relationship between emotional exhaustion and secondary traumatic stress among these groups.
